# Iron Status in Elderly Women Impacts Myostatin, Adiponectin and Osteocalcin Levels Induced by Nordic Walking Training

**DOI:** 10.3390/nu12041129

**Published:** 2020-04-17

**Authors:** Jakub Kortas, Ewa Ziemann, Dariusz Juszczak, Katarzyna Micielska, Marta Kozłowska, Katarzyna Prusik, Krzysztof Prusik, Jedrzej Antosiewicz

**Affiliations:** 1 Department of Sport, Gdansk University of Physical Education and Sport, 80-336 Gdansk, Poland; prusikkatarzyna@gmail.com (K.P.); krzysztof.prusik@awfis.gda.pl (K.P.); 2 Department of Athletics, Strength and Conditioning, Poznan University of Physical Education, 61-871 Poznan, Poland; ziemann.ewa@gmail.com; 3 Seventh Navy Hospital, 80-305 Gdansk, Poland; d.juszczak@7szmw.pl; 4 Department of Anatomy and Anthropology, Gdansk University of Physical Education and Sport, 80-336 Gdansk, Poland; katarzyna.micielska@awfis.gda.pl; 5 Department of Physiology and Pharmacology, Gdansk University of Physical Education and Sport, 80-336 Gdansk, Poland; marta.kozlowska@awfis.gda.pl; 6 Department of Bioenergetics and Physiology of Exercise, Medical University of Gdansk, 80-210 Gdansk, Poland

**Keywords:** ferritin, serum iron, training adaptation, aging

## Abstract

Impaired iron metabolism is associated with increased risk of many morbidities. Exercise was shown to have a beneficial role; however, the mechanism is not well understood. The purpose of this study was to assess the relationship between exerkines and iron metabolism in elderly women before and after 12 weeks of Nordic Walking (NW) training. Exerkines like myostatin, adiponectin, and osteocalcin have been shown to have several positive effects on metabolism. Thirty-six post-menopausal women (66 ± 5 years old, mean ± SD) were randomly assigned to a NW intervention group (n = 18; body mass, 68.8 ± 11.37 kg; fat, 23.43 ± 7.5 kg; free fat mass, 45.37 ± 5.92 kg) or a control group (n = 18; body mass, 68.34 ± 11.81 kg; fat, 23.61 ± 10.03 kg; free fat mass, 44.73 ± 3.9 kg). The training was performed three times a week for 12 weeks, with the intensity adjusted to 70% of the individual maximum ability. Before and one day after the 12-weeks intervention, performance indices were assessed using a senior fitness test. Blood samples (5 mL) were obtained from the participants between 7 and 8 AM, following an overnight fast, at baseline and one day immediately after the 12-week training program. A significant and large time × group interaction was observed for iron (NW: 98.6 ± 26.68 to 76.1 ± 15.31; CON: 100.6 ± 25.37 to 99.1 ± 27.2; *p* = 0.01; $\eta_{p}^{2}$ = 0.21), myostatin (NW: 4.42 ± 1.97 to 3.83 ± 1.52; CON: 4.11 ± 0.95 to 4.84 ± 1.19; *p* = 0.00; $\eta_{p}^{2}$ = 0.62), adiponectin (NW: 12.0 ± 9.46 to 14.6 ± 10.64; CON: 12.8 ± 8.99 to 11.9 ± 8.53; *p* = 0.00; $\eta_{p}^{2}$ = 0.58), and osteocalcin (NW: 38.9 ± 26.04 to 41.6 ± 25.09; CON: 37.1 ± 33.2 to 37.2 ± 32.29; *p* = 0.03; $\eta_{p}^{2}$ = 0.13). Furthermore, we have observed the correlations: basal ferritin levels were inversely correlated with changes in myostatin (*r* = −0.51, *p* = 0.05), change in adiponectin, and change in serum iron (*r* = −0.45, *p* = 0.05), basal iron, and osteocalcin after training (*r* = -0.55, *p* = 0.04). These findings indicate that iron modulates NW training-induced changes in exerkine levels.

## 1. Introduction

The health effects of exercise are well documented. Although the positive impact of exercise has been recently explained, some mechanisms are still not well understood. Exercise-induced changes in iron metabolism are a potential important mechanism of the pro-health effects of exercise. According to an increasing number of reports, impairment of iron metabolism and excessive tissue accumulation of iron may trigger pathological processes. High iron stores are associated with insulin resistance, increased risk of cancer, dysfunction of β-cell, and many others diseases [1,2,3,4]. Exercise training influences iron metabolism and reduces iron accumulation. Iron stores in highly trained athletes and the elderly who participate in recreational activities are lower than those in inactive individuals [5,6].

Excess intracellular iron is stored in ferritin. This protein protects the cell from iron toxicity since ferritin iron does not participate in free-radical–generating reactions. Hence, exposure to increased concentrations of “free iron” or the labile iron pool, i.e., iron that is loosely bound to low-molecular weight compounds such as amino acids, nucleotides, etc., results in an adaptive increase of ferritin biosynthesis [7]. Tissue and serum ferritin levels are correlated. Therefore, serum ferritin is considered to be a good measure of body iron stores in a healthy individual [8].

Exercise training reduces the amount of stored iron via two putative mechanisms. On the one hand, exercise increases the demand for the synthesis of iron-containing proteins. On the other hand, exercise upregulates hepcidin synthesis, inhibiting intestinal iron absorption [9,10]. The final effect of exercise is an adaptive response that relies on reduced iron ferritin stores, which are manifested by decreased serum ferritin levels. Since high serum ferritin levels are associated with an increased risk of many morbidities, their reduction by exercise is desirable.

Another aspect of the health effects of exercise is related to endocrine function of the skeletal muscle and other tissues. Proteins and other molecules liberated into the blood during exercise are called exerkines, and those released by the skeletal muscle are called myokines [11]. Exerkines and myokines exert heterogeneous functions. For example, osteocalcin, liberated from the osteoblast, influences bone metabolism as well as the metabolism of the skeletal muscle, adipose tissue, and other tissues [12]. Most myokines positively affect the metabolism of the skeletal muscle and other tissues. For example, interleukin 6 (IL-6), released by the skeletal muscle during exercise, stimulates lipolysis in the adipose tissue, glycogenolysis in the skeletal muscle, and the synthesis of anti-inflammatory cytokines, such as interleukin 10 (IL-10) [13]. In addition, increased release of osteocalcin and adiponectin by the osteoblast and adipocyte, respectively, during exercise stimulates fat oxidation and improves insulin sensitivity [14]. By contrast, the skeletal muscle-derived myostatin (MSTN), a negative regulator of the skeletal muscle size that inhibits insulin signaling, induces inflammation, and is associated with bone demineralization [15,16]. Moreover, another novel discovered myokine-decorin can act in a paracrine manner and control bone formation as well as muscle hypertrophy by inhibiting myostatin [17]. Blocking MSTN activity by MSTN propeptide overexpression prevents the development of diet-induced obesity and insulin resistance in transgenic animals [15]. Similar to MSTN, high body iron stores are associated with skeletal muscle atrophy, ageing, low bone mass, insulin resistance, and inflammation [18]. Excess tissue iron accumulation may lead to oxidative stress, as iron participates in free-radical–generating reactions. This suggests that the amount of iron accumulated in the tissue may be linked the endocrine function of the tissue. In addition, by acting via the transferrin receptor 2 (Tfr2), serum iron can influence the function of osteoblast and other cells [19].

Exercise induces changes in iron metabolism, reduces serum MSTN levels, and increases osteocalcin and adiponectin levels. However, the interdependence between iron metabolism and these proteins has not been studied in detail. In the current study, we aimed to investigate the association between Nordic Walking (NW) training-induced changes in the circulating levels of MSTN, adiponectin, decorin, and osteocalcin, and their relationship with iron metabolism in elderly subjects.

## 2. Materials and Methods 

### 2.1. Participants

**Figure 1:**
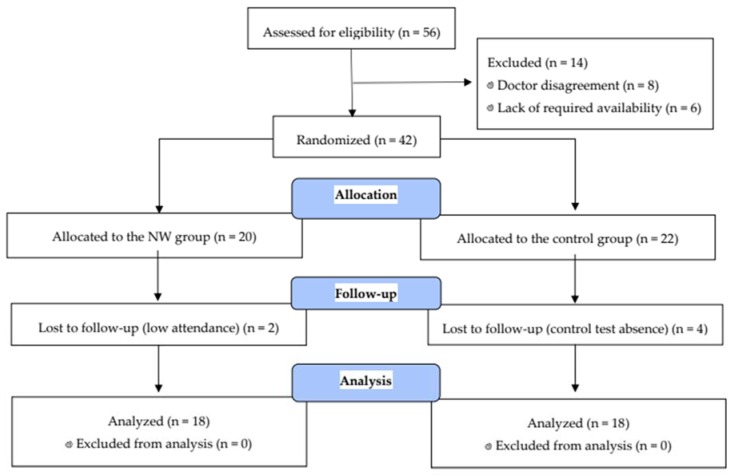
Flow diagram of the study.

The participants were recruited by advertising in the local media and everyday meeting places (churches, bus stations, senior clubs, restaurants, etc.). Fifty-six post-menopausal, elderly women have qualified for the examination. The physical examination took place at the Gdańsk University of Physical Education and Sport and the medical check was performed at the Medical University of Gdansk. The medical check identified women without uncontrolled hypertension (diastolic blood pressure over 100 mmHg), history of cardiac arrhythmia, cardio-respiratory disorders, and orthopedic issues. They were included in the study. Only subjects without any of those contraindications were included in the study (Figure 1) [20].

Women who passed the initial screening were assigned a number corresponding to the project application number. The participants were randomly divided into two groups: the NW group (n = 20) and the control group (CON, n = 22). Randomization for assignment to the experimental groups was performed using an online randomization tool (GraphPad QuickCalcs software, online version), and using the base of the application number.

During the 12 weeks of study, six women were lost to follow-up (minimum required attendance in NW group 80%; in CON group, control test absence). The final participant set included 36 women with an average age of 66.4 ± 4.8 (mean ± SD) years. Eighteen women from the control group were only tested twice (before and after the 12 weeks of study), and did not participate in health training. The control group received the advice to maintain their routine diet and physical activity as previously. They declared not to participate in any regular exercise.

The examination was officially approved by the Bioethical Committee of the Regional Medical Society in Gdańsk (KB-34/18) and the study was performed in agreement with the Declaration of Helsinki. Before commencing the training and testing, the experiment was verbally described to the subjects. The participants were informed that their personal data would be used only during qualification and examination in the current study. Written informed consent was obtained from all participants.

### 2.2. Exercise Protocol and Diet

Before the start of the experiment, the participants attended a meeting covering the training procedure and dietary recommendations. The training schedule was based on a published program [21,22].

The participants met three times a week, 1 h after a light breakfast, and performed the main session of NW training (10-min warm-up, 45–55-min NW, and 10-min cool-down) at 60–70% intensity of their maximum heart rate. Once a week, each participant received a sport-tester device used for current cardiovascular control (Polar, M200). Twelve weeks of exercise (36 training units) were divided into three microcycles. During the first microcycle (six training units), the basic functional efficiency increased, especially the chest mobility and flexibility of the arms and shoulders. Proper walking technique with poles was demonstrated and practiced during that period. The second microcycle (24 training units) was an essential component of the program. Its aim was to improve endurance. It was implemented by a gradual increase of volume (expressed in km walked), which was inherent to increasing the training intensity. In addition, strength and postural muscle-strengthening exercises were performed. The last microcycle (six training units) was an attempt to raise the endurance level by intensifying activity and walking at the fastest possible pace. Further strength exercises, mainly of the back muscles, were performed.

The participants were advised to follow a balanced diet and adhere to healthy eating practices (i.e., consumption at regular time intervals and proper hydration). None of the women consumed a low-iron diet (i.e., vegetarian or vegan). The participants were advised not to change their original dietary habits, but rather to focus on balancing the diet.

### 2.3. Measurements of Body Composition and Physical Fitness

The body mass and composition were determined using a multi-frequency impedance plethysmograph body composition analyzer (In Body 720, Biospace, Korea). The body mass was determined after an overnight fasting, 12 h after any meal or drink. The impedance of body part (trunk, arm, and legs segments) was measured at six different frequencies (1, 5, 50, 250, 500, and 1000 kHz) using an eight-polar tactile-electrode.

The senior fitness test developed by Rikli and Jones [23] for examining elderly people was used to determine the functional fitness of the participants. It consists of six items: (1) 30-s chair stand, (2) arm curl, (3) chair sit-and-reach, (4) back scratch, (5) 8-foot up-and-go, and (6) 2-min step. The items were tested in this order, with 1-min rest between them. Before each item was tested, the evaluator demonstrated the exercise and the participant had an attempt at familiarization, except for the 2-min step test, which the subjects performed only once. The test was conducted twice in the experimental group, after the recruitment and after 12 weeks of training, while the control group was tested only at baseline.

### 2.4. Blood Analysis

At baseline and 1 day immediately after the 12-week training program, blood samples (5 mL) were obtained from the participants, between 7 and 8 AM, following an overnight fast. The serum was obtained by sample centrifugation at 10,009 × *g* for 15 min and stored at −80 °C until analysis. 

Red blood cells count [10^6^⋅μL^−1^] (RBC), haematocrit [%] (Hct), and blood haemoglobin concentration (g⋅dl^−1^) (Hb) were determined from the venous blood samples by conventional methods using a BIOSYSTEMS S.A (Costa Brava, Barcelona, Spain).

The average intra-assay coefficient of variability (CV) was <10% for all assessments. Glucose was measured with a Cobos 6000 analyzer (ROCHE); the serum ferritin level was determined by using SYSMEX XE 2100. Insulin was assessed using immunoassay kit from DiaMetra (catalog no DKO076) within intra-assay CV ≤5% and the inter-assay CV ≤10%.

Serum MSTN level was evaluated using ELISA kits (R&D Systems, USA, cat. no. DGDF80), in accordance with the manufacturer’s instructions. Serum osteocalcin level was evaluated using ELISA kits (Takara, Europe, cat. no. MK114). An enzyme immunoassay method using commercially available kits from Phoenix Pharmaceuticals was employed to determine the plasma adiponectin (catalogue no. EK-ADI-01). The maximal intra-assay CV was 5%. Detection sensitivity was 5.32 pg·mL^–1^. Plasma decorin level was quantified using Human Decorin DuoSet ELISA and DuoSet Ancillary Reagent Kit 2 (R&D Systems, cat. no. DY143 and DY008, respectively) according to the manufacturer ’s protocol. Concentration of parathormon was assessed using the ELISA kit (Demeditec Diagnostics GmbH, cat.no. DE3645) and the sensitivity was 1.57 pg·ml.

### 2.5. Statistical Analysis

Data were given as means with standard deviations (SD). Statistical analyses were performed by using a statistics software package (Statistica 13.1 software).

In the first stage of the analysis, it was checked to see if there were no differences between the groups at baseline in anthropometric, morphological, and physical fitness characteristics. For normal distribution results, an unpaired *t*-test analysis was performed to identify significantly different results at baseline. For the remaining results, the Mann–Whitney test was used.

In this proposal, iron level has been chosen as the main reason for determination of the calculation sample size, based on a clinically relevant improvement of 10% following the training. As a result, at least 12 participants were included in each group (α = 0.05, β = 0.9).

Analysis of changes in the physical performance, body composition, and blood indicators induced by 12 weeks of NW training were performed: for normally-distributed variables, a paired *t*-test; and for others, a Wilcoxon test.

Then, separate 2 (group: INT, CON) x 2 (time: PRE, POST) repeated measures analyses of variances (rANOVA) were calculated [24]. In case of a significant time x group interaction, for homogenous results, Tukey’s post hoc tests for equal sample sizes were performed to identify significantly different results. For heterogeneous results, ANOVA Friedman’s test and Dunn-Bonferroni post-hoc test were used. The effect size (partial eta squared, $\eta_{p}^{2}$) was also calculated, with $\eta_{p}^{2}$ ≥0.01 indicating a small effect, ≥0.059 indicating a medium effect, and ≥0.138 indicating a large effect [25]. The relationships between variables were evaluated using the Spearman correlation coefficient. The level of significance was set at *p* < 0.05.

## 3. Results

### 3.1. General Outcomes

The baseline anthropometric and morphological characteristics of the participants are summarized in Table 1. 

We measured physical fitness level, which demonstrated no significant differences between CON and the NW group at baseline (Table 1). The 12 weeks of NW training resulted in no significant changes in body composition and morphology (Table 2). All the physical fitness components improved. However, statistically significant differences were observed for four items: chair stand (*p* = 0.05, CI: 0.01–3.14) and 2-min step (*p* = 0.04, CI: 2.18–30.18) in the endurance test, and chair sit-and-reach (*p* = 0.00, CI: 3.3–9.49) and back scratch (*p* = 0.03, CI: 0.19–1.76) in the flexibility test (Table 2).

### 3.2. Biochemical Changes

**Figure 2:**
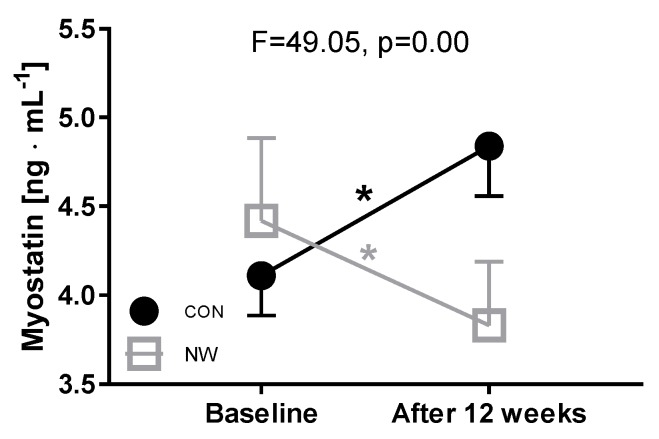
Changes in MSTN levels after 12 weeks, * *p* < 0.05.

NW training significantly decreased the MSTN levels. A significant and large time × group interaction was observed (*p* = 0.00; $\eta_{p}^{2}$ = 0.62), with post-hoc comparisons indicating a significant decrease in the NW group from PRE to POST (–13%, *p* = 0.00), and a significant increase in CON (18%, *p* = 0.00) (Figure 2).

In addition, the decrease in MSTN levels was inversely associated with baseline ferritin levels of the participants who took part in training (*r* = −0.51, *p* = 0.05, Table 3). Correlation between all measured variables in the NW group are shown in the Supplementary Material. 

Serum iron and ferritin levels for both groups are shown in Table 4. A significant and large time × group interaction was only observed for serum iron levels (*p* = 0.01; $\eta_{p}^{2}$ = 0.21), with post-hoc comparisons indicating a greater decrease in the NW group from PRE to POST than that in the CON group (NW: –23%, *p* = 0.00; CON: –1%, *p* = 0.98). No differences in ferritin concentration after 12 weeks of the experiment were observed in either group.

NW training significantly increased the adiponectin levels. A significant and large time × group interaction was observed (*p* = 0.00; $\eta_{p}^{2}$ = 0.58), with post-hoc comparisons indicating a greater increase in the NW group from PRE to POST than that in the CON group (NW: 22%, *p* = 0.00; CON: –7%, *p* = 0.12). Further, the adiponectin level increase after the training was inversely correlated with a decrease in serum iron levels (*r* = −0.45, *p* = 0.05). In addition, NW training induced a significant increase in the osteocalcin levels. A significant and moderate time × group interaction was observed (*p* = 0.03; $\eta_{p}^{2}$ = 0.13), with the post-hoc comparisons indicating a greater increase in the NW group from PRE to POST than that in the CON group (NW: 7%, *p* = 0.02; CON: 0.3%, *p* = 0.99). Of note, osteocalcin levels after 12 weeks of NW were correlated with the pre-intervention serum iron levels (*r* = −0.55, *p* = 0.04). The increase in decorin level after the training was not significant, but it inversely correlates with significant decrease in myostatin.

## 4. Discussion

Previously, we have shown that NW training decreases body iron stores, which could be considered as an adaptive and pro-health response to exercise [26]. In the present study, for the first time, we demonstrated that NW training induces the decrease in serum MSTN levels and is inversely correlated with baseline serum ferritin levels in elderly women. The skeletal muscle is one of the tissues that accumulate iron. The total iron stored there is comparable with the amount stored by the liver [27]. In the current study, the amount of iron stored in the skeletal muscle has not been determined. However, it can be assumed that NW endurance training reduces its amount. Previously, we have reported that 12 weeks of NW training significantly improves endurance in elderly women [22], and this observation was confirmed herein. Conversely, it is well documented that adaptation to the endurance exercise is associated with an increased synthesis of such skeletal muscle proteins as myoglobin, cytochrome, and some enzymes, all of which require iron for physiological function [10]. Therefore, it can be expected that their increased synthesis augments demand for iron, which can lead to diminished ferritin accumulation in tissues. In our previous study, we applied 12 or 32 weeks of NW training exercises [22,26]. For the first time, we did not observe significant changes in ferritin after 12 weeks training. Possibly, in this case, longer intervention is needed.

The relationship between iron metabolism and MSTN has not been studied to date. MSTN-induced skeletal muscle degradation is mediated by the inhibition of Akt kinase activity, which can lead to the activation of FOXO3a transcriptional activity and increased expression of atrogin 1 [28]. Recently, it has been shown that downregulation of Akt leads to iron accumulation in the skeletal muscle in an animal model [29]. In addition, circulating ferritin levels correlate with insulin resistance in women and men [30]. Insulin resistance of the skeletal muscle is associated with reduced activity of Akt kinase [28], and low Akt activity leads to the activation of FoxO1, which increases the *MSTN* mRNA levels in C_2_C_12_ myotubes. Taken together, the data suggest that an increased accumulation of iron in tissues, such as the skeletal muscle, heart, and adipose tissue, may negatively modulated their endocrine function by lowering Akt activity and increasing MSTN synthesis. Poor exercise-induced decrease in MSTN levels in women with higher serum ferritin levels observed in the current study confirms this hypothesis. Further, according to several studies, different types of exercise induce changes in the serum MSTN levels [31,32,33], and these levels are inversely correlated with the skeletal muscle function in elderly women [34]. The data presented herein suggest that changes induced by the training are modulated by the iron store status. Interestingly, in the CON group, the serum MSTN levels increased after 12 weeks and did not correlate with ferritin levels. The current study was performed in the winter, which is usually associated with reduced physical activity and could augment MSTN synthesis. In addition, it is important to note that the brown adipose tissue, which expands in response to cold temperature, is an important source of MSTN [35]. These data suggest that NW training could be a good natural remedy to prevent the rise of MSTN levels in winter.

Another exerkine whose synthesis can be modified by iron is adiponectin. Adiponectin is a hormone that modulates a number of metabolic processes, including insulin sensitivity and fatty acid oxidation [36]. Serum ferritin levels are inversely correlated with adiponectin levels [37]. Data from the current study did not confirm this, however, as the correlation was not observed at baseline when all subjects were analyzed together. Conversely, adiponectin levels increased after the NW training and inversely correlated with changes in serum iron levels. In animal studies and in an experimental cell culture model, iron negatively regulates adiponectin transcription via FOXO1-mediated repression [38]. Another putative mechanism responsible for a NW training-induced shift in adiponectin levels involves elevated osteocalcin levels. An uncarboxylated form of osteocalcin, liberated from the osteoblast, can act as a hormone that triggers the release of adiponectin from the adipose tissue [39]. According to several studies, exercise training increases osteocalcin levels [14,40]. The current study revealed that the serum osteocalcin levels after 12 weeks of NW training inversely correlates with baseline serum iron levels. Iron downregulates osteocalcin gene expression in vitro [41], supporting this observation. The applied training program did not alter the decorin concentration in elderly women. Still, to the best of authors knowledge, this is the first study that shows the impact of NW training on circulating decorin in regard to MSTN status.

The signaling role of iron is well recognized as iron induces the formation of reactive oxygen species and modulates several signaling pathways, e.g., stress-activated protein kinase, nuclear factor kappa-light-chain-enhancer of activated B cells (NF-κB), and other pathways [42]. Iron triggers signaling through Tfr2 expressed in the liver, erythroblast, and osteoblast [19]. Tfr2 signaling triggered by transferrin saturated with iron in the osteoblast likely impedes bone formation [41]. As mentioned above, exercise training modulates iron metabolism by reducing tissue iron stores, both in highly trained athletes and in recreationally active elderly people [5,6]. This indicates that the reduction of body iron stores to an optimal level is an important part of the adaptation process to exercise. 

Exerkines and myokines play important roles in exercise-induced pro-health changes in the human body. Collectively, the presented and published data indicate that stored iron, represented by serum ferritin, and serum iron both affect the impact of NW training on exerkine synthesis. Further studies are needed to understand the mechanistic details of the effect of iron status on MSTN, adiponectin, and osteocalcin levels. 

## Figures and Tables

**Table 1:** Anthropometric, morphological, and physical fitness characteristics of participants.

	NW (*n* = 18)	CON (*n* = 18)	*p*	95% CI
Age (years)	66.78 ± 4.76	66.12 ± 4.83	0.55	(−1.67; 1.54)
Body mass (kg)	68.8 ± 11.37	68.3 ± 11.81	0.91	(–8.71; 7.79)
BMI (kg·m^-2^)	25.87 ± 3.45	25.96 ± 5.12	0.95	(–2.97; 3.14)
Fat (kg)	23.4 ± 7.5	23.6 ± 10.03	0.95	(–6.05; 6.41)
Fat (%)	33.4 ± 6.8	33.3 ± 9.45	0.95	(–5.94; 5.62)
*FFM (kg)*	*45.4 ± 5.92*	*44.7 ± 3.9*	*0.65*	*(–4.28; 3)*
*SMM (kg)*	*24.6 ± 3.58*	*24.3 ± 2.31*	*0.59*	*(–2.46; 1.92)*
VFA (cm^2^)	133.2 ± 25.39	128.8 ± 35.98	0.69	(–26.21; 17.46)
Hb (g∙dL^–1^)	13.9 ± 0.81	14.0 ± 0.88	0.80	(–0.52; 0.67)
Ht (%)	42.8 ± 2.64	41.6 ± 2.28	0.18	(–2.88; 0.56)
WBC (G∙L^–1^)	5.6 ± 1.55	6.4 ± 1.45	0.14	(–0.27; 1.83)
MCH (pg)	30.7 ± 1.15	30.3 ± 1.24	0.31	(–1.26; 0.42)
MCHC (g∙dL^–1^)	32.5 ± 1.03	33.6 ± 0.73	0.00	(0.44; 1.67)
Chair Stand (n)	20.67 ± 4.4	19.28 ± 4.31	0.37	(−4.49; 1.71)
*Arm Curl (n)*	*19.4 ± 19.1*	*24.67 ± 14.46*	*0.26*	*(−4.18; 14.72)*
2-Min Step (n)	132.07 ± 24.95	133.39 ± 23.85	0.88	(−16.04; 18.69)
*Chair Sit-&-Reach (cm)*	*6.57 ± 7.49*	*4.42 ± 6.37*	*0.56*	*(−9.53; 5.24)*
*Back Scratch (cm)*	*7.77 ± 10.82*	*3.19 ± 6.02*	*0.14*	*(−10.65; 1.51)*
8-Foot Up-&-Go (s)	3.74 ± 0.82	3.69 ± 0.53	0.82	(−0.54; 0.43)

Values are means ± SD. The statistical significance level was obtained using: italics—Mann–Withney test; normal font—unpaired *t*-test. Fat, fat mass; FFM, free fat mass; SMM, skeletal muscle mass; VFA, visceral fat area; Hb, hemoglobin; Ht, hematocrit; WBC, white blood cells; MCH, mean cell hemoglobin; MCHC, mean cellular hemoglobin concentration; NW, Nordic Walking group; CON, control group; 95% CI, 95% confidence interval of differences between study groups at baseline.

**Table 2:** Changes in the physical performance, body composition, and blood indicators induced by 12 weeks of NW training.

	NW (*n* = 18)			
	PRE 12 weeks	POST 12 weeks	*p*	95% CI
Chair stand (n)	20.7 ± 4.4	22.3 ± 4.64	0.05	(0.01; 3.14)
*Arm curl (n)*	*19.4 ± 19.1*	*19.9 ± 18.19*	*0.53*	*(−0.99; 3.13)*
2-Min step (n)	132.1 ± 24.95	146 ± 23.3	0.04	(2.18; 30.18)
*Chair sit-and-reach (cm)*	*6.6 ± 7.49*	*11.7 ± 7.76*	*0.00*	*(3.3; 9.49)*
*Back scratch (cm)*	*7.8 ± 10.82*	*8.7 ± 10.46*	*0.03*	*(0.19; 1.76)*
8-Foot up-and-go (s)	3.7 ± 0.82	3.6 ± 0.73	0.52	(−0.59; 0.32)
Fat (kg)	23.4 ± 7.5	22.2 ± 7.82	0.72	(−2.09; 1.48)
Fat (%)	33.4 ± 6.8	32.1 ± 6.97	0.75	(−2.62; 1.93)
*FFM (kg)*	*45.4 ± 5.92*	*45.5 ± 6.1*	*0.65*	*(−1.5; 1.87)*
Hb (g∙dL^−1^)	13.9 ± 0.81	13.9 ± 0.87	0.81	(−0.26; 0.2)
Ht (%)	42.8 ± 2.64	42.2 ± 2.47	0.11	(−1.77; 0.21)
WBC (G∙L^−1^)	5.6 ± 1.55	5.8 ± 1.91	0.77	(−0.77; 1.02)
MCH (pg)	30.7 ± 1.15	30.5 ± 1.14	0.22	(−0.51; 0.42)
MCHC (g∙dL^−1^)	32.5 ± 1.03	32.9 ± 0.91	0.16	(−0.31; 1.07)

Values are means ± SD. The statistical significance level was obtained using: italics—Wilcoxon test; normal font—paired *t*-test. Fat, fat mass; FFM, free fat mass; Hb, hemoglobin; Ht, hematocrit; WBC, white blood cells; MCH, mean cell hemoglobin; MCHC, mean cellular hemoglobin concentration; NW, NW group; CON, control group; 95% CI, 95% confidence interval of differences between pre and post values in the training group.

**Table 3:** Significant Spearman correlation between variables in NW group.

Variables	*r*	*p*
Change in MSTN vs. baseline ferritin	−0.51	0.05
Change in MSTN vs. baseline iron	0.03	0.36
Change in MSTN vs. change decorin	−0.55	0.04
Change in adiponectin vs. change in ferritin	0.41	0.08
Change in adiponectin vs. change in iron	−0.45	0.05
Baseline iron vs. osteocalcin after 12 weeks	−0.55	0.04

**Table 4:** Changes in iron metabolism, exerkines, and myokines levels.

	NW (*n* = 18)		CON (*n* = 18)		ANOVA	
	PRE 12 weeks	POST 12 weeks	PRE 12 weeks	POST 12 weeks	Group × time	η_p_^2^
Iron (µg∙dL^–1^)	98.6 ± 26.68	76.1 ± 15.31 *	100.6 ± 25.37	99.1 ± 27.2	0.00	0.21
Ferritin (ng∙mL^–1^)	107.8 ± 58.2	104.6 ± 53.85	113 ± 56.36	116.9 ± 51.7	0.15	0.06
Glucose (mg∙dL^–1^)	89.4 ± 23.77	93.2 ± 6.13	103.6 ± 7.57	95.2 ± 22.84	0.21	0.05
Insulin (µU∙mL^–1^)	7.6 ± 4.89	8.5 ± 4.08	8.5 ± 5.49	8.4 ± 5.23	0.17	0.06
Decorin (ng∙mL^–1^)	10.7 ± 4.04	11.7 ± 4.1	10.5 ± 1.68	10.6 ± 1.81	0.49	0.01
Osteocalcin (ng∙mL^–1^)	38.9 ± 26.04	41.6 ± 25.09 *	37.1 ± 33.2	37.2 ± 32.29	0.03	0.13
Parathormon (pg∙mL^–1^)	48.7 ± 7.01	50.7 ± 10.21	48.4 ± 13.9	46.3 ± 13.9	0.05	0.11
Adiponectin (μg∙mL^–1^)	12.0 ± 9.46	14.6 ± 10.64 *	12.8 ± 8.99	11.9 ± 8.53	0.00	0.58

Values are means ± SD. * differences between PRE and POST values in the group.

## Supplementary Materials

The following are available online at https://www.mdpi.com/2072-6643/12/4/1129/s1, Table S1: Values of Spearman correlation between all measured variables in NW group. I—baseline values, II—values after 12 weeks, Δ—changes after training. Bold values are statistically significant, *p* < 0.05.

## Limitations

Failing to ensure that the participants in the control group have followed the same diet represents limitations of the study. Thus, to assess if a dietary regimen can modify the exercise-induced iron response, further research should entail effective dietary monitoring even in a control group. Despite of this limitation, observed interdependent myokines and iron metabolism is important value of this paper.
