# Supplementary material for: Iron Status in Elderly Women Impacts Myostatin, Adiponectin and Osteocalcin Levels Induced by Nordic Walking Training
Source: Nutrients. 2020 Apr 17;12(4):1129. doi: 10.3390/nu12041129 (PMC7231223; doi:10.3390/nu12041129)
Supplement: Supplementary file 1 [file nutrients-12-01129-s001.pdf]

Supplementary 1. Values of Spearman correlation between all measured variables in NW group. I – baseline values, II – values after 12 weeks, Δ – changes after training. Bold values are statistically significant, $p <. 0.05$

	I iron [µg/dl]	II iron [µg/dl]	Δ iron [µg/dl]	I ferritin [ng/dl]	II ferritin [ng/dl]	Δ ferritin [ng/dl]	I glucose [mg/dl]	II glucose [mg/dl]	Δ glucose [mg/dl]	I insulin [µU/mL]	II insulin [µU/mL]	Δ insulin [µU/mL]	I decorin [ng/mL]	II decorin [ng/mL]	Δ decorin [ng/mL]	I osteocalcin [ng/mL]	II osteocalcin [ng/mL]	Δ osteocalcin [ng/mL]	I parathormon [pg/mL]	II parathormon [pg/mL]	Δ parathormon [pg/mL]	I adiponectin [ng/mL]	II adiponectin [ng/mL]	Δ adiponectin [ng/mL]	I myostatin [pg/mL]	II myostatin [pg/mL]	Δ myostatin [pg/mL]
I iron [µg/dl]	X	**0,57**	**-0,80**	0,27	0,25	0,06	-0,08	0,07	0,11	**0,53**	**0,51**	-0,30	0,01	-0,21	-0,37	-0,33	**-0,55**	-0,20	0,00	0,13	0,29	-0,38	-0,30	0,13	-0,19	-0,29	0,03
II iron [µg/dl]		X	-0,11	-0,06	-0,13	0,29	-0,17	-0,11	0,04	**0,46**	0,18	**-0,49**	0,09	0,00	-0,20	-0,21	-0,26	0,10	-0,30	-0,15	0,07	**-0,52**	**-0,54**	-0,14	-0,02	-0,03	-0,03
Δ iron [µg/dl]			X	-0,09	-0,17	-0,13	-0,11	-0,26	-0,03	-0,27	**-0,46**	-0,01	0,01	0,24	0,34	0,33	**0,45**	0,25	-0,22	-0,13	-0,13	0,03	-0,11	**-0,45**	0,12	0,27	0,07
I ferritin [ng/dl]				X	**0,93**	-0,32	0,26	0,15	-0,35	0,39	0,42	0,02	-0,05	0,28	0,38	0,25	0,10	0,01	0,01	0,30	0,22	0,15	0,09	-0,03	0,34	0,18	**-0,51**
II ferritin [ng/dl]					X	-0,05	0,37	0,19	**-0,49**	0,33	0,43	0,06	0,18	0,28	0,23	0,31	0,12	-0,16	0,20	0,38	0,16	0,14	0,11	0,08	0,28	0,17	-0,45
Δ ferritin [ng/dl]						X	-0,07	-0,18	-0,03	-0,20	-0,17	0,15	**0,57**	0,03	-0,41	0,11	-0,02	-0,42	0,07	-0,15	-0,31	-0,17	-0,12	0,41	-0,38	-0,17	0,25
I glucose [mg/dl]							X	**0,88**	**-0,81**	**0,55**	**0,49**	-0,41	-0,05	0,05	0,07	0,14	0,17	0,36	0,41	**0,60**	0,33	-0,32	-0,36	-0,24	-0,12	-0,35	-0,19
II glucose [mg/dl]								X	**-0,50**	**0,61**	**0,50**	**-0,51**	-0,10	0,04	0,08	-0,08	-0,09	0,40	0,19	0,32	0,26	-0,26	-0,28	-0,13	-0,14	-0,40	-0,17
Δ glucose [mg/dl]									X	-0,41	-0,37	0,32	-0,03	-0,04	-0,04	-0,36	-0,38	-0,21	**-0,58**	**-0,72**	-0,29	0,34	0,36	0,35	-0,09	0,05	0,22
I insulin [µU/mL]										X	**0,68**	**-0,61**	-0,06	-0,08	-0,10	-0,12	-0,27	0,12	0,00	0,08	0,17	**-0,46**	**-0,45**	-0,20	0,09	-0,18	-0,31
II insulin [µU/mL]											X	0,03	-0,10	-0,14	0,03	0,03	0,00	0,11	0,15	0,30	0,32	-0,30	-0,33	-0,31	0,11	-0,21	-0,43
Δ insulin [µU/mL]												X	-0,13	-0,14	0,15	0,10	0,31	-0,04	0,01	-0,03	-0,12	0,34	0,33	0,01	-0,05	0,00	-0,07
I decorin [ng/mL]													X	**0,59**	0,00	0,08	-0,06	-0,31	-0,03	-0,05	0,02	-0,13	-0,13	0,38	-0,25	-0,24	0,24
II decorin [ng/mL]														X	**0,74**	0,08	0,06	-0,01	-0,17	0,11	0,25	0,31	0,25	**0,47**	0,15	0,05	-0,26
Δ decorin [ng/mL]															X	0,06	0,19	0,21	-0,19	0,09	0,21	0,42	0,31	0,09	0,41	0,23	**-0,55**
I osteocalcin [ng/mL]																X	**0,88**	0,06	0,26	0,02	-0,34	0,17	0,07	-0,26	-0,01	0,17	0,18
II osteocalcin [ng/mL]																	X	**0,47**	0,35	0,22	-0,18	0,12	0,06	-0,35	-0,14	0,00	0,24
Δ osteocalcin [ng/mL]																		X	0,11	0,27	0,14	-0,11	-0,15	-0,34	-0,17	-0,16	0,16
I parathormon [pg/mL]																			X	**0,55**	-0,09	-0,05	0,03	-0,04	-0,06	-0,04	0,13
II parathormon [pg/mL]																				X	**0,74**	-0,28	-0,25	-0,13	0,08	-0,02	-0,20
Δ parathormon [pg/mL]																					X	-0,36	-0,38	-0,13	0,06	-0,14	-0,28
I adiponectin [ng/mL]																						X	**0,97**	**0,48**	0,40	**0,46**	-0,28
II adiponectin [ng/mL]																							X	**0,60**	0,38	0,44	-0,26
Δ adiponectin [ng/mL]																								X	0,00	0,02	-0,09
I myostatin [pg/mL]																									X	**0,89**	**-0,81**
II myostatin [pg/mL]																										X	**-0,53**
Δ myostatin [pg/mL]																											X
